# Hybrid deep learning approach to improve classification of low-volume high-dimensional data

**DOI:** 10.1186/s12859-023-05557-w

**Published:** 2023-11-07

**Authors:** Pegah Mavaie, Lawrence Holder, Michael K. Skinner

**Affiliations:** 1 School of Electrical Engineering and Computer Science, Washington State University, Pullman, WA 99164 USA; 2 School of Biological Sciences, Center for Reproductive Biology, Washington State University, Pullman, WA 99164-4236 USA

**Keywords:** Deep learning, Machine learning, Hybrid learning, Representation learning, Classification

## Abstract

**Background:**

The performance of machine learning classification methods relies heavily on the choice of features. In many domains, feature generation can be labor-intensive and require domain knowledge, and feature selection methods do not scale well in high-dimensional datasets. Deep learning has shown success in feature generation but requires large datasets to achieve high classification accuracy. Biology domains typically exhibit these challenges with numerous handcrafted features (high-dimensional) and small amounts of training data (low volume).

**Method:**

A hybrid learning approach is proposed that first trains a deep network on the training data, extracts features from the deep network, and then uses these features to re-express the data for input to a non-deep learning method, which is trained to perform the final classification.

**Results:**

The approach is systematically evaluated to determine the best layer of the deep learning network from which to extract features and the threshold on training data volume that prefers this approach. Results from several domains show that this hybrid approach outperforms standalone deep and non-deep learning methods, especially on low-volume, high-dimensional datasets. The diverse collection of datasets further supports the robustness of the approach across different domains.

**Conclusions:**

The hybrid approach combines the strengths of deep and non-deep learning paradigms to achieve high performance on high-dimensional, low volume learning tasks that are typical in biology domains.

## Introduction

With the progress of machine learning (ML) in the past few decades, ML has become a prominent solution for different applications including image classification [1], text mining [2], bioinformatics [3, 4], and activity recognition [5]. Learning accurate models requires generation of informative features. In many datasets, this process is labour intensive and requires significant domain knowledge to identify relevant features. In the case of high-dimensional data, feature generation is even more challenging due to the high computation cost of evaluating the potentially exponential number of different feature sets. Another challenge in many settings (e.g., genomic datasets) is class imbalance due to the low frequency of phenomena of interest (e.g., disease states). Fewer training examples of the class of interest further complicates the learning process. One commonly-accepted relationship between sample size *n* and number of features or dimensions *d* to avoid overfit is for $d<\sqrt{n}$ [6]. For the datasets used in this work (see Table 1), $d>\sqrt{n}$, so the use of a smaller number features will tend to improve classification performance.

**Table 1:** Summary of datasets and best predictive model

Dataset	#Samples	#Classes	#Features	Best predictive model	Best accuracy	Class frequency
WISDM	27,452	6	320	Hybrid (1-block, layer: 5)	0.793	Walk 4588
						Upstairs 4416
						Downstairs 4438
						Sit 4678
						Stand 4698
						Jog 4634
HAR	10,299	6	561	Hybrid (5-blocks, layer: 10–12)	0.925	Walk 1722
						Upstairs 1544
						Downstairs 1406
						Sit 1777
						Stand 1906
						Lie 1944
Amazon	1000	2	100	Hybrid (3-blocks, layer: 5)	0.797	Review > 2 500
						Review ≤ 2 500
Yelp	1000	2	100	Hybrid (3-blocks, layer: 2)	0.776	Review > 2 500
						Review ≤ 2 500
IMDb	748	2	100	Hybrid (3-blocks, layer: 5)	0.797	Review > 2 374
						Review ≤ 2 374
Gene Promoter	106	2	57 × 4	Hybrid (3-blocks, layer: 1)	0.952	Promoter 53
						Non-promoter 53

For each dataset, the number of available samples, number of classes, and number of features are shown. The Gene Promoter features use a one-hot encoding of 57 base-pairs, each one of four values [a, c, g, t]. The Best Predictive Model is shown along with the number of blocks and the feature extraction layer. The Best Accuracy for this model is also shown. The class frequencies are given in the last column

Deep learning (DL) as a part of machine learning has improved the predictive model performance since the early 2000s [7] by automatically extracting, analysing, and understanding useful information directly from the raw data. A major strength of deep learning networks (DNNs) is their ability to generate increasingly complex features as the inputs to one layer are combined using a variety of functions (e.g., convolution) and passed to the next layer. The result is a set of highly complex features that are used to perform the learning task. One of the advantages of deep learning over machine learning is that it does not require manually extracted or handcrafted features and it tries to learn the data representation as part of the training phase.

On the other hand, using DL has its own challenges when it comes to the training of the network. First, DL networks usually require a large amount of data to train a strong classifier, compared to traditional ML algorithms. This is because the number of parameters that need to be learned is much higher than most other learning algorithms. Second, DL requires significant hyperparameter tuning. Many of these hyperparameters are controlling the training of a DL model, and finding the best settings can take a considerable amount of time compared to other ML approaches.

**Fig. 1:**
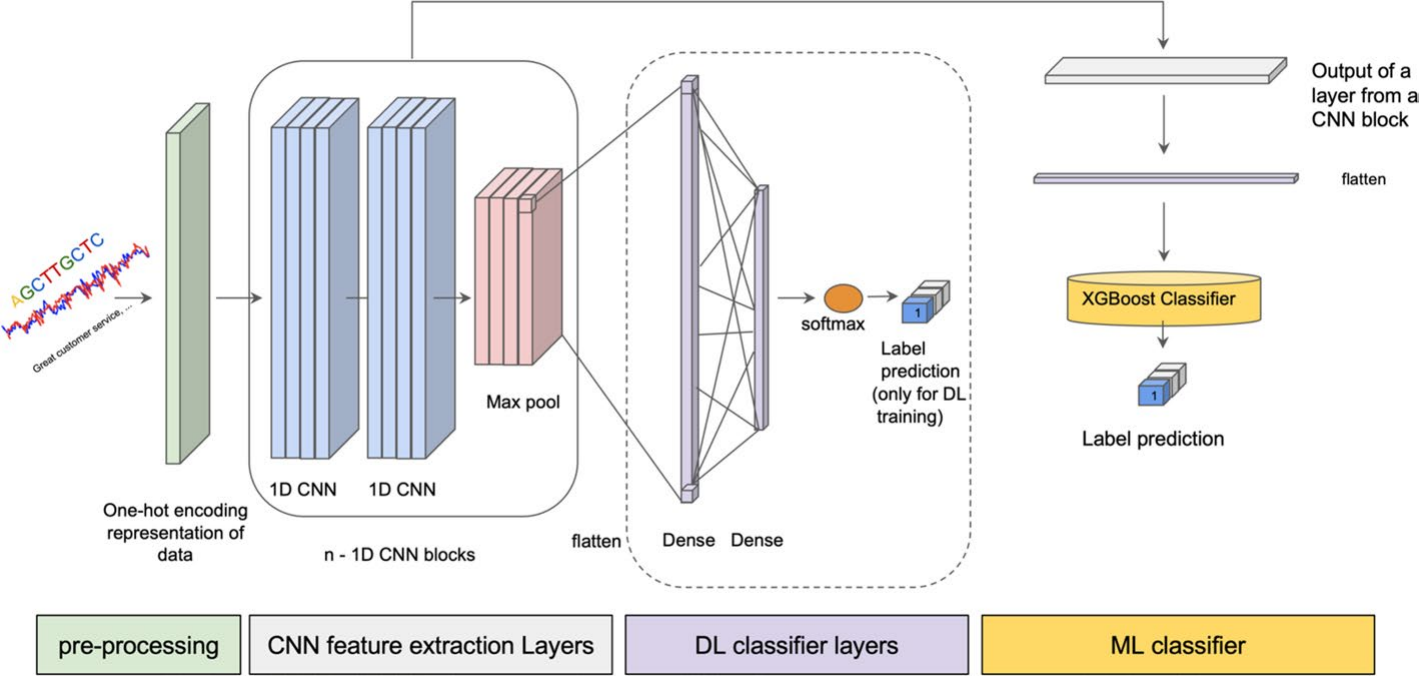
Simplified diagram of the hybrid model. The raw features provided with the datasets are input to the DNN. Real-valued features are input directly, and categorical features are input using a one-hot encoding. Each CNN block consists of two CNN layers followed by a max pooling layer. Features are extracted from a CNN layer. The CNN blocks are followed by a flattening layer and two dense layers leading to the classification. The labeled examples are used for training the network, but the network is not used for the final label prediction, as indicated by the dashed box around the DL classifier layers. Features extracted from the CNN layer are input to the XGBoost ML classifier for training and final predictions

The main contribution of this work is a method for combining deep and non-deep learning methods into a hybrid method illustrated in Fig. 1 that outperforms the individual methods. The method proceeds by training a supervised DNN for feature extraction for the targeted classification task and using the extracted feature representation from the DNN for training a traditional ML classifier. This approach takes advantage of learning a data representation from raw data using DL methods. In addition, feeding the learned data representation to the ML classifier helps to decrease the demand of having large amounts of data for training the classifier. The hybrid approach can also help to increase the interpretability of the DL representation of the data. This is based in part on the increased interpretability of the classifications made by decision-tree-based classifiers, like XGBoost, and its ability to rank features by importance [8]. Interpretability of the DL features was also demonstrated by earlier work with the hybrid approach on epigenetic data in which DNA motifs based on the important XGBoost features were visualized [4]. The results on six different domains show that this hybrid approach can outperform DL or ML alone.

Data representation is a crucial factor of the performance in most ML methods [9]. For that reason, much of the actual effort in applying ML methods goes into the design of feature extraction, preprocessing and data transformation steps. Representation learning is the acquisition of data representations that facilitate the extraction of relevant information for building classifiers and predicting outcomes. When using probabilistic models, a suitable representation commonly encompasses the posterior distribution of the explanatory factors that influence the input data. An appropriate representation also serves as input for a supervised predictor [10]. Among feature extraction algorithms, Principal Components Analysis or PCA is one of the oldest and most widely used approaches [11, 12]. Unfortunately, the expressive power of linear features is limited; they cannot be stacked to form deeper, more abstract representations since the composition of linear operations yields another linear operation. Goodfellow et al. [13] have found that distributed and sparse representations are the typical ways to achieve more expressiveness compared to non-sparse representations such as PCA.

Hinton et al. [14] used a hierarchical set of features, using an unsupervised representation learning method to learn a transformation, where in each iteration of unsupervised representation learning, the method stacks one layer of weights to a deep neural network. Then, the set of layers could be used as a combination for initializing a deep supervised predictor, such as a neural network classifier, or a deep generative model, such as a Deep Boltzmann Machine [9, 10, 15].

The topological structure of input dimensions, such as the layout of pixels in images, the structure of videos, and the sequential structure of text, can be utilized to define local receptive fields. These fields allow for computing low-level features from a subset of the input, with a sparse weight matrix and non-zeros only allowed for topologically local connections. The convolutional network is based on this idea and has been used for object recognition and image segmentation. The architecture of convolutional networks is argued to be used by mammalian brains for object recognition [16].

The representation extracted from the DL network can be fed into another neural network classifier, or it can be used as features in a traditional ML classifier. Incorporating a traditional ML classifier can be helpful when there are limited examples for training a DL network. Results show that feature extraction from earlier layers in the DL network typically outperforms feature extraction from the last layer of the DL network, which is a common technique in hybrid approaches. Therefore, this hybrid approach can reduce the number of learning parameters and the complexity of the networks.

## Related work

Previous work has shown that a hybrid approach can improve performance in several domains. Tsai and Wang [17] combine a neural network and a decision tree to predict stock prices. Wan et al. [18] replace the final layer of a neural network with decision trees, called neural-backed decision trees, which improves performance on image recognition tasks and takes advantage of the interpretability of decision tree classifiers. Kong and Yu [19] use a random forest decision tree classifier to identify relevant features in the sparse learning scenario, where the number of features exceeds the number of training examples. The features are then input to a deep neural network to avoid overfitting. Their approach improved performance on gene expression classification tasks. Kontschieder et al. [20] use random forest decision trees to initially route data to an appropriate subnetwork, which reduces the variance in the data before classification by the network. Their approach achieved state-of-the-art performance on several image classification tasks. Grover et al. [21] use a hybrid approach to improve performance on modeling a set of weather variables. Their approach uses the outputs of traditional predictors of the variables as input to a neural network to refine those predictions. Wang et al. [22] linearly combine the predictions of three models (neural network, support vector regression, and decision tree) to predict the outcomes of optimizations to a plasma arc process for reforming tar. Qaid et al. [23] develops deep- and transfer-learning techniques to detect COVID19 using Convolutional Neural Networks (CNNs), transfer learning, and ML techniques. Akhtar et al. [24] use a CNN for generating a set of optimized features and use a Support Vector Machine (SVM) as the classifier.

Other hybrid approaches have combined CNNs with the XGBoost classifier, as proposed here, but by adding XGBoost after the last layer of the CNN, which does not consider feature extraction from an earlier layer. Thongsuwan et al. [25] combine the two methods by adding XGBoost as the last layer of the CNN and show that the combined approach outperforms either method alone on several datasets. However, their method considers only the last layer of the CNN from which to extract features, but our results show that the last layer is usually not the best layer for feature extraction. Ren et al. [26] also combine the two methods by adding XGBoost after the last layer of the CNN and show performance competitive with other hybrid and non-hybrid approaches on two benchmark image datasets. Zivkovic et al. [27] combine the two methods by adding XGBoost after the last of three dense layers in the CNN. Their approach does perform some parameter optimization, including the number of CNN layers, which effectively varies the last layer from which features are extracted, but without training the CNN along with additional deeper layers, as in our approach. Their approach showed superior performance on a COVID-19 X-ray image dataset. Li et al. [28] use features for XGBoost extracted from the output of a linear layer after the CNN layers. However, one novelty to their approach is the combination of features from the second CNN block along with the last layer, which together are input to the final linear layers of the CNN. Their approach outperformed competing approaches, including CNN and XGBoost alone, on a social media prediction task of user interest in posted images.

The hybrid approach has demonstrated success in numerous domains by combining the strengths of deep learning and traditional machine learning methods. Several combination approaches have been used, including sequential staging of the underlying methods, ensembles of parallel predictions, and integration of one method inside another. The hybrid approach proposed here is novel in that the traditional ML model is used as the basis for predictions, but the features used by the ML model are extracted from an internal layer of a DL model trained to perform the same prediction task independent of the ML model training.

In this work, we propose a hybrid model for a general classification task that yields better performance in most of the cases compared to standalone ML or DL models. The hybrid approach trains a DL network to extract a data representation from the raw data and uses the extracted features for training an ensemble-based ML classifier. The model is used for supervised learning tasks, so the extracted data representation is with respect to the labels of the classification task.

## Results

**Fig. 2:**
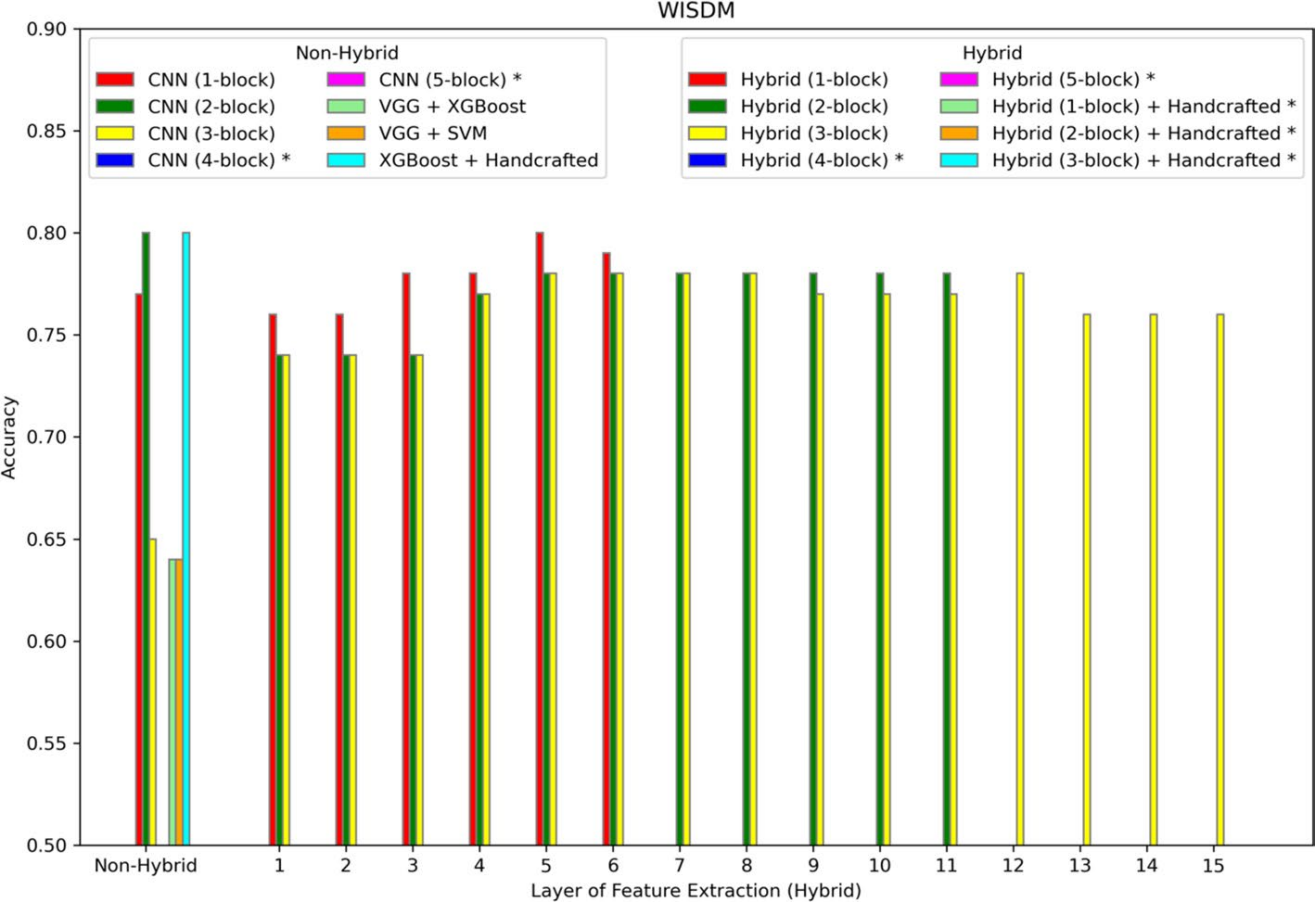
Accuracy of different models on the WISDM dataset. The first position along the X-axis shows the accuracy of all the non-hybrid models. The remaining positions show the performance of the hybrid models, where features are extracted from different layers of the hybrid network. Asterisks in the legend indicate models not applied to this dataset

**Fig. 3:**
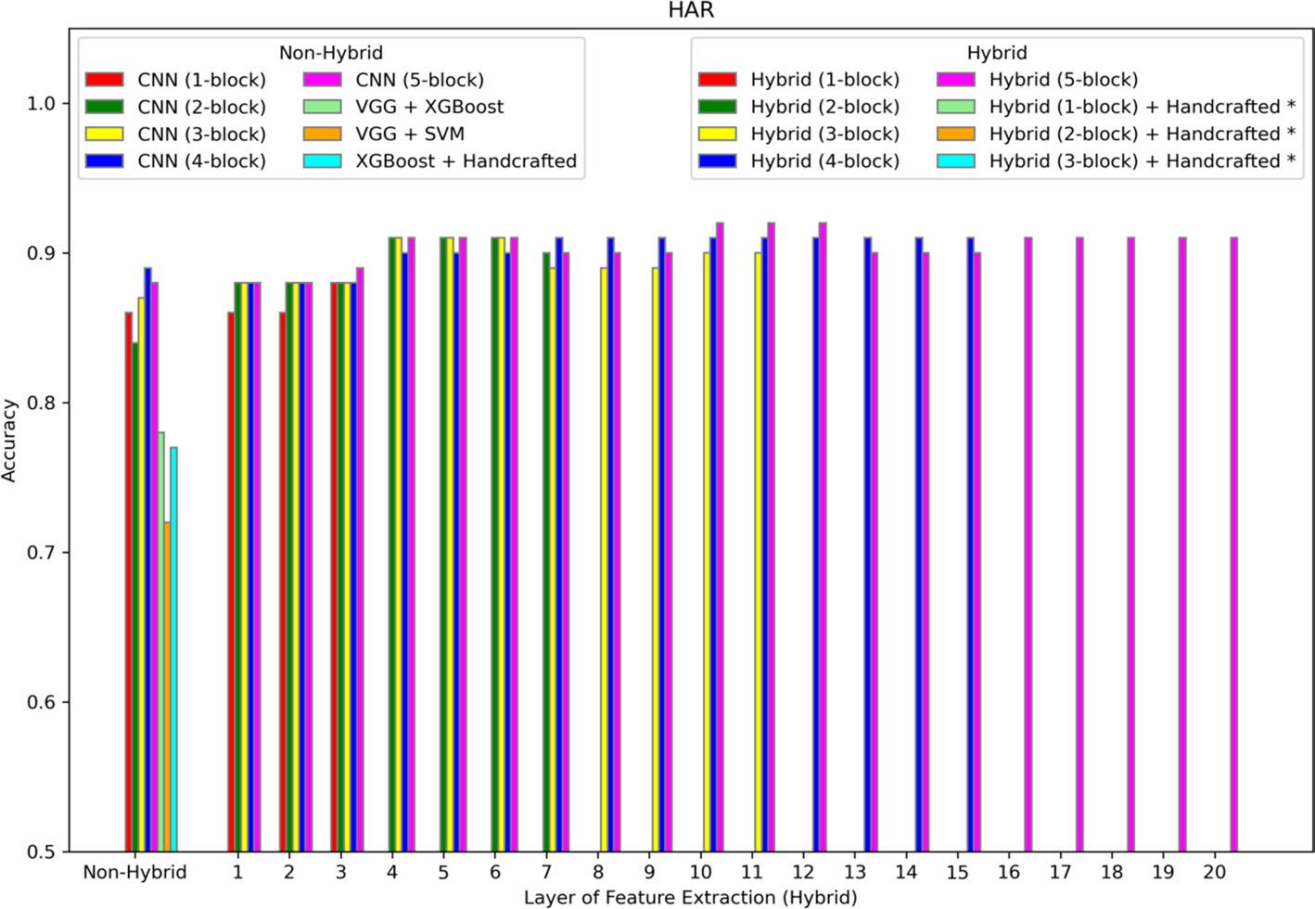
Accuracy of different models on the HAR dataset. The first position along the X-axis shows the accuracy of all the non-hybrid models. The remaining positions show the performance of the hybrid models, where features are extracted from different layers of the hybrid network. Asterisks in the legend indicate models not applied to this dataset

**Fig. 4:**
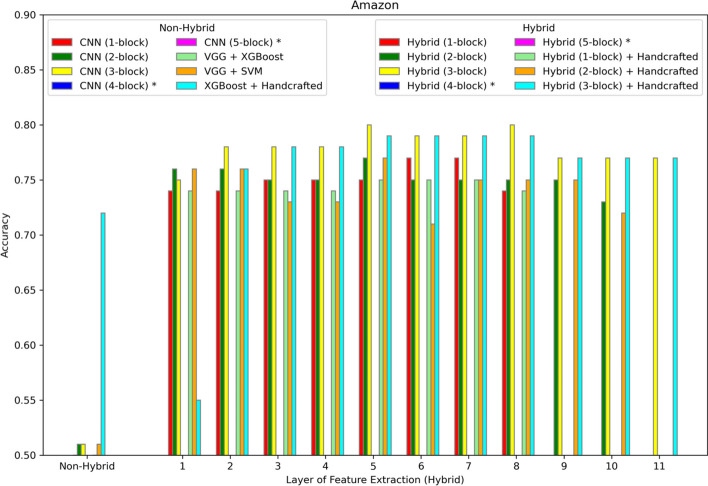
Accuracy of different models on the Amazon dataset. The first position along the X-axis shows the accuracy of all the non-hybrid models. The remaining positions show the performance of the hybrid models, where features are extracted from different layers of the hybrid network. Asterisks in the legend indicate models not applied to this dataset

**Fig. 5:**
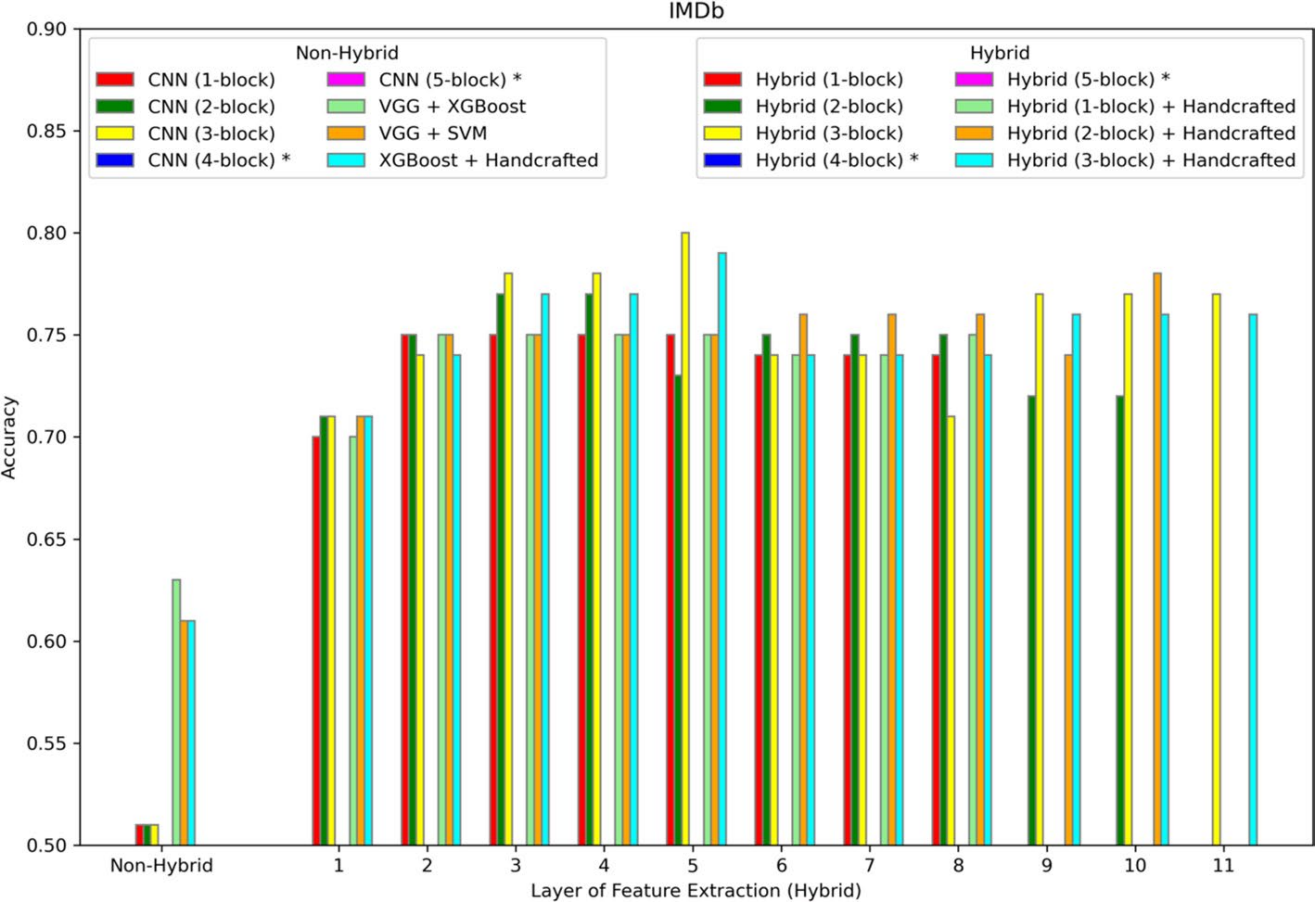
Accuracy of different models on the IMDb dataset. The first position along the X-axis shows the accuracy of all the non-hybrid models. The remaining positions show the performance of the hybrid models, where features are extracted from different layers of the hybrid network. Asterisks in the legend indicate models not applied to this dataset

**Fig. 6:**
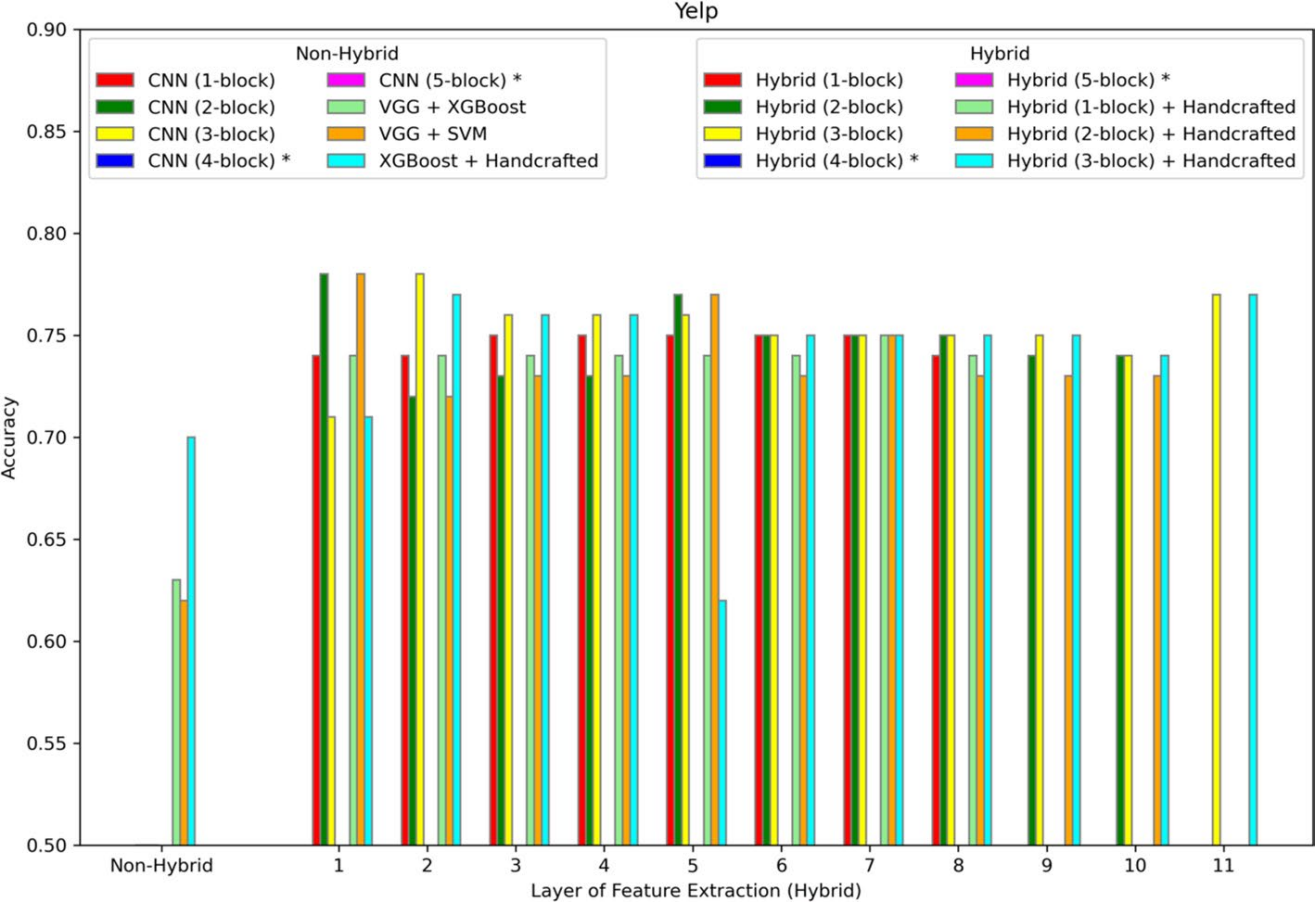
Accuracy of different models on the Yelp dataset. The first position along the X-axis shows the accuracy of all the non-hybrid models. The remaining positions show the performance of the hybrid models, where features are extracted from different layers of the hybrid network. Asterisks in the legend indicate models not applied to this dataset

**Fig. 7:**
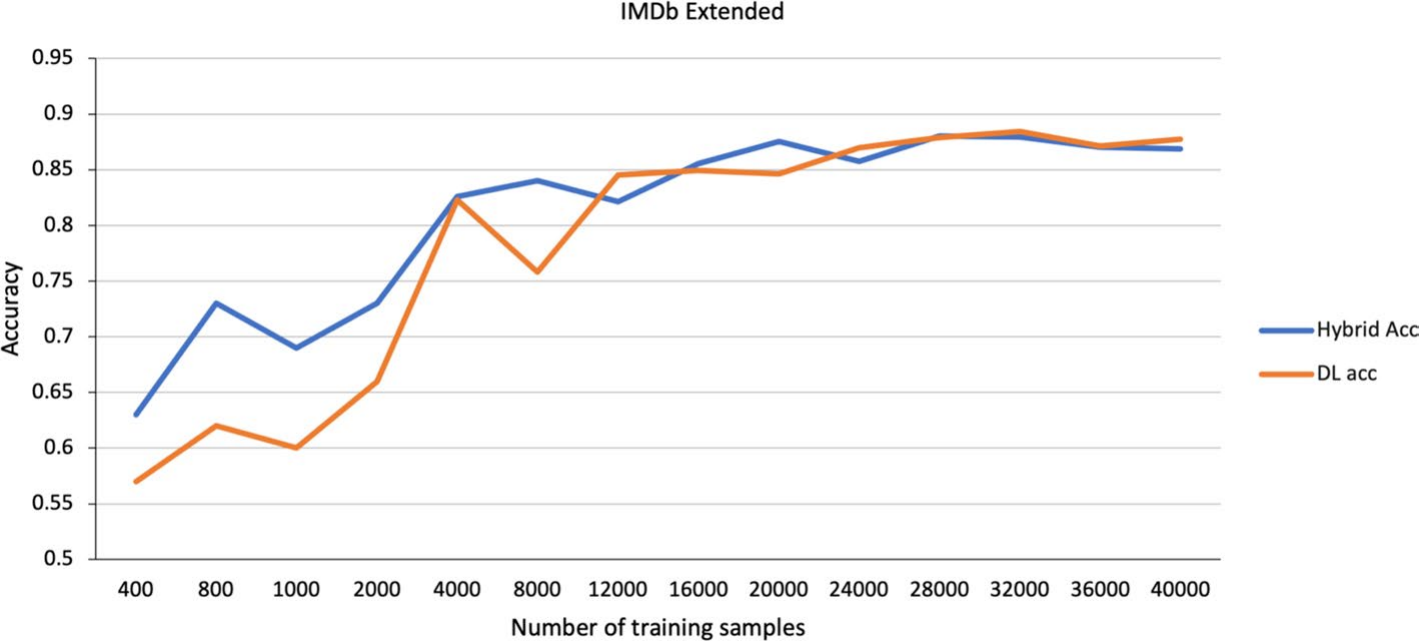
Performance of different models on the larger IMDb Extended dataset using different size training samples. The hybrid model achieves better accuracy at lower sample sizes, but the DL-alone model does achieve hybrid-level performance with sufficient samples

**Fig. 8:**
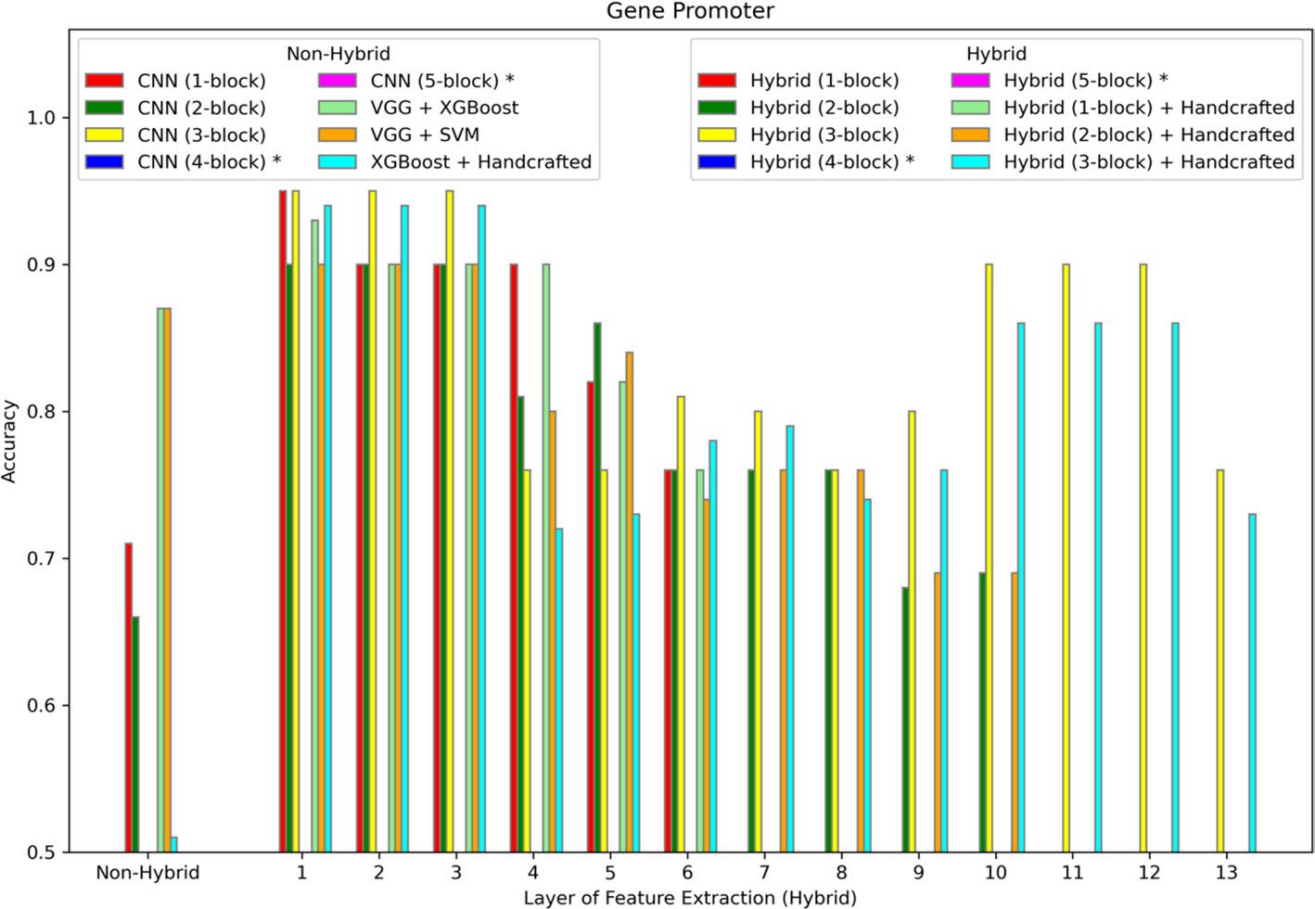
Accuracy of different models on the Gene Promoter dataset. The first position along the X-axis shows the accuracy of all the non-hybrid models. The remaining positions show the performance of the hybrid models, where features are extracted from different layers of the hybrid network. Asterisks in the legend indicate models not applied to this dataset

The hybrid model is evaluated on six different datasets summarized in Table 1 and described in more detail in the Methods section. The results are shown in Figs. 2, 3, 4, 5, 6, 7 and 8. Three different aspects of the hybrid model performance are assessed: (1) The performance of the predictive models, (2) the ability to add handcrafted features to the ML classifier besides CNN extracted representation, and (3) the reliability of the hybrid model on larger datasets. The first scenario measures the quality of the hybrid approach using different data representations. The accuracy score is used to evaluate the performance of the predictive models using different sets of features. The models are trained using 80% of the dataset, and then the accuracy is computed by testing the model on the remaining 20%. The accuracy metric is used due to the generally balanced class distribution of the datasets, as shown in Table 1. The CNN model is used for feature extraction as a benchmark classifier to evaluate the power of the hybrid learner. Also, two different hybrid approaches are used as benchmark classifiers. Qaid et al. [23] uses a pre-trained Visual Geometry Group (VGG) model as the feature learner and an XGBoost as a classifier (VGG + XGBoost), and we use another hybrid model that uses a VGG for data representation and an SVM as the classifier (VGG + SVM). These benchmarks are modified to make the models applicable to these datasets. In addition, the parameters of the CNN are tuned to optimize performance. In iterative steps, convolutional blocks are added to the CNN, the model is retrained, and features are extracted from all the possible options to measure their effects on the performance. The addition of CNN blocks is terminated when accuracy no longer improves. Finally, the accuracy of the hybrid model is reported on the different datasets.

Another ability of the hybrid approach that can be assessed is that the handcrafted features can be used in the ML classifier alongside the CNN-extracted features. Therefore, to evaluate the impact of adding handcrafted features, we compute the accuracy score for different CNN-extracted features with handcrafted features or without them using XGBoost and compare the accuracy of the model using those different types of features.

The other motivation for the hybrid approach was that using an ML classifier reduces the demand for having extensive data samples that traditional DL models need to perform well. So, for better insight into the performance of the hybrid method, one can gradually increase the number of training samples and monitor the accuracy until the CNN model chosen as our benchmark matches the performance of the hybrid model. For this experiment, a larger dataset with more training samples is needed, in this case, an extended version of the internet movie database (IMDb) dataset with 50,000 samples. The training sample size provided to each method is increased from 400 to 40,000 while monitoring the CNN accuracy and the hybrid accuracy. For choosing the right data representation from the CNN model, features are extracted from all the possible layers, XGBoost is then trained using each representation, and the best accuracy score is reported.

### Results for activity recognition models

Figure 2 shows the results for the WISDM dataset. The most informative data representation is extracted from the output of the sixth layer. Also, the best option for the feature learner model is a one-block CNN network. We increase the number of blocks for each model until the accuracy is not improved. The best feature representation for these activity recognition datasets lay on the upper layers which represent more high-level features. For this dataset, the DL-alone model with three blocks achieves similar accuracy to the hybrid model, but this occurs only for this dataset.

Figure 3 shows the performance of different models on the HAR dataset. The hybrid model outperforms other models. The best performance results from the features input to XGBoost that are extracted from the tenth layer of a five-blocks CNN model.

### Results for sentiment analysis models

For sentiment analysis, Figs. 4, 5 and 6 show the results for Amazon, IMDb, and Yelp respectively. In each case the best performance is achieved by the hybrid approach with the three-block CNN and with features extracted from the seventh layer. In addition, the same experiments show that adding handcrafted features to the hybrid approach does not improve accuracy.

Figure 7 shows how increasing the number of training samples affects the performance of the DL-alone and hybrid models for the extended IMDb dataset. The DL-alone performance increases with sample size. This is because the DL-alone model has 4.3 million trainable parameters, and increasing the number of training samples results in a more robust classifier. As shown in the Fig. 7, the DL-alone and hybrid performance are comparable at larger sample sizes.

### Results for genomic model

Figure 8 shows that the hybrid models far exceed the performance of other models, including XGBoost with handcrafted features, whose accuracy is 0.51, and three-block CNN, whose accuracy is 0.47 and therefore not shown on the plot. Best performance is achieved by the hybrid approach using a three-block CNN and extracting features from one of the first three layers.

## Discussion

Overall, the results show that the hybrid approach outperforms the DL network alone and the non-deep learning XGBoost ML approach. Furthermore, the use of handcrafted features either as additional inputs to the hybrid approach or with XGBoost does not improve performance. This indicates that the features extracted from the DL network are superior to the handcrafted features. Yet the DL network trained alone, though it is using the same features, performs worse than the hybrid approach. This is due to the small sizes of the training sets (low volume). But as Fig. 7 shows, the performance of the DL network alone will likely reach the hybrid performance given enough training data.

The best predictive hybrid model for each dataset described in Table 1 yields some insights into the decision of the number of blocks to include in the hybrid model and the layer from which to extract the features. The WISDM dataset, having the highest number of training examples, needs only a one-block network to achieve best performance, but still a higher (deeper) layer from which to extract complex features built from the original 320 features. As observed in Fig. 2, the two-block CNN DL-alone network is still capable of achieving top performance without the hybrid approach. For the remaining datasets having fewer examples than the WISDM dataset, the hybrid approach is superior. The best hybrid models for the last four datasets in Table 1 require fewer blocks because they have fewer features; whereas the HAR dataset requires more blocks due to the much higher feature count. The WISDM dataset defies this trend due to the much higher sample size. Therefore, the number of blocks resulting in the best performance may be correlated with the number of features, as higher numbers of features require more blocks when fewer samples are available. Similarly, the feature extraction layer is deeper with higher dimensional datasets indicating that more complex features are needed to properly represent the data. The number of blocks in the best hybrid model may also be influenced by the number of classes in the dataset. Two-class problems are generally easier than six class problems, assuming similar sample volume and dimensionality. So, the two-class problems tend to require few blocks (less deep networks), although this trend is confounded by the varying numbers for samples and features.

Simpler feature generation methods, like PCA, may provide similar performance to the hybrid approach in some cases, but the goal here is to avoid having different methods for different datasets. For example, in the case of the Gene Promoter results in Fig. 8, while the best performing features are extracted from early layers, the features from these layers are still complex, non-linear features compared to the linear orthogonal features computed by PCA. Also, PCA is an unsupervised method; whereas, the hybrid approach learns features in a supervised setting. So, the features will be more correlated to the class rather than merely maximizing variance. Still, a comparison between CNN-XGBoost and PCA-XGBoost would help to identify when, and to what extent, the complexity of the CNN is needed.

Although an advantage of the hybrid approach is less dependence on handcrafted features, performance on the HAR dataset compared to the WISDM dataset provides a good example of the benefits of handcrafted features. Both datasets have the same classes and collect similar low-level sensor data. But the HAR dataset uses derived features (total acceleration, body acceleration, total angular velocity, and both time and frequency domain features). The WISDM dataset uses only the original acceleration and rotation features. Accuracy on the HAR dataset (Fig. 3) is generally higher in the 89%-92% range compared to the similar task on the WISDM dataset with accuracies ranging from 75 to 80% (Fig. 2). Still, the hybrid accuracy at 92% was able to improve upon the 89% accuracy of the non-hybrid DL-alone (four-block CNN). In the other four datasets (Amazon, IMDb, Yelp, Gene Promoter), models with and without handcrafted features are compared directly. In each case, the best hybrid model without handcrafted features outperforms both the hybrid and non-hybrid models with handcrafted features. Therefore, the hybrid approach can improve performance whether the data is represented using raw or handcrafted features, which reduces the user’s burden for both manually generating features and choosing the best type of machine learning model for the task.

## Conclusions

The hybrid learning approach extracts features from a deep network and uses them within a non-deep learning method to perform classification. Results from several domains show that this hybrid approach outperforms standalone deep and non-deep learning methods. Hybrid models were trained with different parameters to find the best set of data representations. Even though the feature extraction layer resulting in the best performing model varies across different datasets, the results show that extracting features from higher levels can perform well. Table 1 shows the summary of all the datasets and the performance of the best models. Results show that there is at least one data representation that outperforms an ML or DL baseline model for all tested datasets.

Since the best model in each dataset varies in terms of the size of the underlying DL network and the layer from which features are extracted, a next step in this work is to identify a method for estimating these optimal parameters based on properties of the dataset. More generally, there is a need to identify the types of datasets for which the hybrid model is superior, especially datasets for which increasing numbers of training examples does not close the performance gap between the hybrid model and DL alone. The initial results indicate that the hybrid model will be superior for small, sequence-based, high-dimensional datasets.

## Methods

Representation learning using a DNN requires a large set of training examples [10]. This is the main motivation for this study to use a DNN as a representation learner and a traditional ML algorithm as a classifier. A DNN is trained on the original training examples, but for the classification task, an ML classifier uses the output from a layer of the DNN as the feature set. The ML model is fit on re-expressed input which is extracted from the DNN. Choosing the right layer for extracting the data representation is vital in the performance of the ML model. The proposed hybrid model shown in Fig. 1 is a general approach that can be used in different classification domains. This framework contains two main components: a Deep Neural Network (DNN) and a traditional ML classifier, described below.

The hybrid approach is evaluated on six datasets summarized in Table 1. Each dataset requires some modifications to the models based on the original features used to describe the data and the number of classes to choose from for classification. Each dataset and the associated modifications are also described below.

### Deep neural network classifier

The role of the DNN component is to learn a representation of the input data by constructing complex features guided by supervised training on the target learning task. The convolutional neural network (CNN) was chosen in this work due to its success in multiple, diverse domains and its strength in generating complex features. CNNs are used in many domains such as image classification [29], object recognition [30], activity recognition [31], and many other applications. CNNs have multiple abstractions of levels that use a non-linear model to transform the original data into higher abstract levels and non-linear functions. The proposed CNNs in this work contain several convolutional blocks and fully connected layers. A CNN has a hierarchical architecture. Starting from the input signal $x$, each subsequent layer ${x}_{j}$ is computed as:

$${x}_{j} = \rho {W}_{j}{x}_{j-1}$$

where $\rho$ is a non-linearity operator and ${W}_{j}$ is a linear operator.

Each convolutional block consists of two convolutional layers followed by a max-pooling layer. These layers serve to generate new complex features based on the input sequence and reduce the dimension of the previous convolutional layer for input into the next convolutional layer. These blocks allow features to be constructed independently of their position in the input sequence. Convolutional layers are using ReLU as their activation function. The output of ReLU function is computed as:

$$Relu (x) = max(0,x)$$

A batch-normalization layer is used after each convolutional layer. This step regulates the value of activation. The learning rate can be safely increased to accelerate the learning process, reduce overfitting, avoid activation function saturation and gradient vanishing, and increase the stability of the network [32]. For each convolutional layer, we need to decide the number of filters and the kernel size. The first convolutional layer does not use padding, but the second one uses padding to conserve the size of the output.

After two convolutional layers, a max-pooling layer is used to reduce the number of features and the spatial dimension of the activation maps without loss of information. It also helps to prevent overfitting. At the end of each block, a dropout layer randomly drops neurons from the network and further helps the network to overcome the overfitting problem by reducing the number of parameters.

After the convolution-max-pooling blocks, there is a classifier block, which contains two dense layers and a classifier layer. Dense layers connect all the neurons in one layer to all neurons of the next layer. Dense layers combine high dimensional feature maps and map them into one dimensional vectors. They also use error back-propagation based on correlation to the final classification. The first dense layer contains 256 nodes, and the second dense layer contains 128 nodes. The classifier layer is a dense layer whose outputs are fed to a SoftMax activation function to make the final classification. The SoftMax function for input vector *z* and *k* classes is computed as:

$$\sigma \left({z}_{i}\right)= \frac{{e}^{{z}_{i}}}{\sum_{j=1}^{k}{e}^{{z}_{j}}}$$

The loss function is binary cross-entropy (BCE) which is computed as:

$$BCE= -(ylog\left(p\right)+\left(1-y\right)\mathrm{log}\left(1-p\right))$$

where $y$ is the binary indicator of the correct classification and $p$ is the predicted probability of the class. To prevent overfitting the validation loss value is monitored. If the value does not decrease after 10 epochs the training process is terminated.

### Machine learning classifier

Traditional ML methods have two advantages over DL methods. First, ML methods typically require significantly fewer training examples than DL methods to achieve similar performance. This is mainly due to ML methods’ inherent representational bias (e.g., decision trees) and requirement for user-provided features that serves to narrow the space of potential ML models compared to the much larger space of potential DL models. However, fewer training examples are needed to learn a representative set of features by the DL model, and these features can then be extracted for use in the ML model; thus, satisfying the requirement for relevant features. Second, traditional ML methods typically provide more interpretation behind a particular classification. For example, a decision tree can provide the sequence of decisions made to reach the final classification.

Another challenge for both ML and DL methods is class imbalance, where one class occurs with much higher frequency than another class in the data. Two main approaches to address class imbalance are bagging and boosting. In the bagging method, multiple models are generated based on random samples of the training data or the feature set. The final classification is based on a majority vote of all the models [33]. Random Forest [34] is one of the best performing bagging methods. Random Forest constructs an ensemble of decision trees, where each tree is built using a random subset of the available features.

The boosting approach to class imbalance also constructs an ensemble of models, but in a sequence, where each model in the sequence is biased to avoid the errors made by the previous model. XGBoost [35] is one of the best performing boosting methods. XGBoost constructs an ensemble of decision trees and uses gradient boosting, where new models are biased toward predicting the gradient in the errors of previous models. Several studies have shown that XGBoost typically outperforms Random Forest, so XGBoost is used here as the ML classifier component of the hybrid approach. As an added benefit, since the member models of an XGBoost ensemble are decision trees, XGBoost can provide a ranking of features based on a feature’s information gain, i.e., ability to partition the data into more homogeneous sets. This importance ranking on features helps to identify which properties of the data are most highly correlated with the classification.

XGBoost was chosen based partly on its superiority on imbalanced problems, since that was an issue in our previous epigenetics dataset [4]. XGBoost was also chosen due to its use of decisions trees that support feature selection, better interpretation of the classifications, and generally good performance on diverse domains. But the class imbalance strength is not exercised in this study to focus on the systematic evaluation of the hybrid nature of the approach. Additional future experimentation including imbalanced datasets would be instructive.

Let us assume that the features for training the XGBoost classifier are extracted from the output of the second convolutional layer. The new representation for the input features $y$ is computed as:

$${y}_{1}=I * {W}_{j}+ {b}_{j } for j=1, 2, \dots , F$$

$${y}_{1}{\prime}=ReLU({y}_{1})$$

$${y}_{2}={y}_{1}{\prime} * {W}_{j}+ {b}_{j } for j=1, 2, \dots , F$$

$${y}_{2}{\prime}=ReLU({y}_{2})$$

where $F$ is the number of filters, ${y}_{i}$ is the output corresponding to the ${j}^{th}$ convolution filter, ${W}_{j}$ is the weights of the ${j}^{th}$ filter, and ${b}_{j}$ is the ${j}^{th}$ bias.

The version of XGBoost used in this work is Python version available at https://github.com/dmlc/xgboost. The default parameters are used with no optimization due to the overall intent to provide a general-purpose hybrid solution that does not rely on optimization per dataset.

### Activity recognition datasets

Data collected from body-worn sensors give scientists a better insight to study human behaviour [5]. But extracting features and data processing for these signal-type data are expensive. Here we consider two activity recognition datasets to evaluate the hybrid approach. The two activity recognition datasets used are HAR and WISDM, summarized in Table 1.

The first dataset for evaluating the performance of the model is the WISDM human activity recognition dataset from University of California at Irvine (UCI) [36]. The full dataset contains accelerometer and gyroscope data collected from 51 test subjects, some from smartphones and some from smartwatches, performing 18 different activities. From this dataset we used just the accelerometer data taken from the smartphone users conducting six of the most frequent activities in the data: sitting, standing, upstairs, downstairs, walking, jogging. For each activity, the acceleration for the x, y, and z-axis was captured with a timestamp. Thirty-six people were involved in the data collection process. The data contains six different labels and 27,452 samples. We split the data into two subsets for this experiment: 80% randomly chosen for training (20% of the training data is for validation), and the remaining 20% for testing. Training sample size is 20,868, and the testing sample size is 6,584. As part of the pre-processing step, we convert all the features into normalized floating-point numbers and convert the string class labels to integers. There are two parameters for creating each sample from continuous data: the number of steps for one time segment (time-period), which is set to 80, and the steps to take from one segment to the next (time-step), which in this experiment is 40. If these two values are equal, then there is no overlap between the segments. The time step of 80 results in 80 * 4 = 320 features per example.

The second chosen data set is Human Activity Recognition (HAR) using smartphones dataset from UCI [36]. The data is collected among a group of 30 volunteers within an age bracket of 19–48 years. Each person wore a smartphone at their waist and performed six different activities: walking, walking downstairs, walking upstairs, standing, sitting, lying down. The raw accelerometer and gyroscope data taken over a 2.56 s time window were processed into 561 features for each example. We split the data into two subsets for this experiment: 80% randomly chosen for training (20% of the training data is for validation), and the remaining 20% for testing. The training samples are 7352 and the testing samples are 2947. In the pre-processing phase, all the features are converted to floating point numbers and normalized. The labels are converted to integers.

The CNN model used for the activity recognition datasets consists of several convolutional blocks and a dense block. For the first convolutional block, 64 filters are used, and the size of each filter is 3. As we add another convolutional block during our experiments, we double the number of filters and keep the kernel size the same. The first convolutional layer does not use padding, but the second one uses padding to conserve the size of the output. The activation function for each convolutional layer is the Rectified Linear Unit (ReLU).

The main network architecture follows the general approach as defined in our earlier work [3, 4]. A batch-normalization layer is used after each convolutional layer. After two convolutional layers, a max-pooling layer is used to generalize the model; the pooling size for the max-pooling layer is 2. At the end of each block, a dropout layer is added to the DNN. The dropout rate is 0.4. After the convolution-max-pooling blocks is the classifier block, which contains a flattening layer, two dense layers, and a classifier layer. The first dense layer contains 256 nodes, and the second dense layer contains 128 nodes. Feature weights using error back-propagation are based on correlation to the final classification. The classifier layer is a dense layer with an output node for the label. SoftMax is used for the activation function. The loss function is binary cross-entropy, and the network optimizer is the Adam optimizer.

### Sentiment analysis datasets

Sentiment analysis, or opinion mining, is the task of finding the opinion of the writer. Sentiment analysis is one of the most popular research areas in natural language processing (NLP) [37]. We evaluate the effectiveness of the hybrid model on three data sets: Amazon, IMDb, and Yelp. These datasets are originally extracted from [38] and summarized in Table 1.

In the pre-processing phase for these datasets, we use the tokenization technique. Tokenization is a method to segregate a particular text into small chunks or tokens. A tokenizer function is used for vectorizing a text corpus [39]. Each text input is converted into a sequence of integers that has a coefficient for each token. The tokenizer function is fitted on the text with 10,000 maximum words, and each sample at most contains 100 words.

The Amazon reviews dataset rates products on a scale from 1 to 5, but the dataset is used as a binary classification dataset [36, 40]. If a review is higher than 2, it will be considered a positive sample, and otherwise, it is a negative sample. The dataset has 1000 samples that are randomly extracted from Amazon reviews, and the label distribution is balanced [36, 38]. We use 80% of the data for training (20% of the training data is for validation) and the remaining 20% for the testing process.

The IMDb movie review sentiment dataset was originally introduced by Maas et al. [41]. The dataset rates movies on a scale from 1 to 5, but the dataset is used as a binary classification problem [36, 38]. If a review is higher than 2.5, it will be considered a positive sample, and otherwise, it is negative sample. The dataset contains 748 samples. We split the data into two subsets for this experiment: 80% randomly chosen for training (20% of the training data is for validation), and the remaining 20% for testing. Another version of the IMDb reviews dataset is used that contains 50,000 data samples [41]. This dataset is used to gradually increase the size of training samples and monitor the performance of the hybrid model comparing to the CNN.

The Yelp reviews dataset [42] contains restaurant reviews on a scale from 1 to 5, but the dataset is used as a binary classification problem. If a review is higher than 2.5, it will be considered a positive sample, and otherwise, it is negative sample. There are 1000 samples [36, 38]. The label distribution is balanced, so there are 500 positive and 500 negative samples. We split the data into two subsets for this experiment: 80% randomly chosen for training (20% of the training data is for validation), and the remaining 20% for testing.

Several modifications were made to the models in order to process the sentiment analysis datasets. Before the convolutional blocks, we add an embedding at the top of the network. Since the input shape is larger and protecting the temporal information can be a problem, we choose each filter’s size as 5. In the first convolutional block, 64 filters are used, and the size of each filter is 5. As we add another convolutional block during our experiments, we double the number of filters and keep the kernel size the same. The activation function for each convolutional layer is “ReLU”. After two convolutional layers, a max-pooling layer is used. The pooling size for the max-pooling layer is 3. At the end of each block, a dropout layer is added to the DNN. The dropout rate is 0.4.

After the convolution-max-pooling blocks is the classifier block, which contains a flattening layer, two dense layers, and a classifier layer. The first dense layer contains 64 nodes, and the second dense layer contains 1 node. The classifier layer is a dense layer with an output node for the label. Sigmoid is used for the activation function. The loss function is binary cross-entropy, and the network optimizer is the RMSprop optimizer.

### Genomic dataset

Machine learning continues to improve our ability to analyze genomic datasets, but typically requires many handcrafted features. Deep learning has also been successfully applied to such datasets but suffers in the face of typically small datasets. Therefore, our hybrid approach is well-suited to such domains. As an example, we evaluate the hybrid approach on a gene promoter dataset [36]. The gene promoter dataset is a small dataset with 106 samples. It is a binary dataset that shows whether a 57 base-pair DNA sequence is a member or non-member of the class of sequences with biological promoter activity. This dataset contains 53 positive instances and 53 negative instances. Each of the 57 base-pairs is one of [a, c, g, t]. We split the data into two subsets for this experiment: 80% randomly chosen for training (20% of the training data is for validation), and the remaining 20% for testing. The dataset properties are summarized in Table 1.

For the genomic dataset the data is one-hot encoded and the input shape for the DNA sequences is 57 × 4. We can reduce the size of filters and choose the filter size as 3. In the first convolutional block, 64 filters are used, and the size of each filter is 3. As we add another convolutional block during our experiments, we double the number of filters and keep the kernel size the same. The activation function for each convolutional layer is “ReLU”. After two convolutional layers, a max-pooling layer is used. The pooling size for the max-pooling layer is 2. At the end of each block, a dropout layer is added to the DNN. The dropout rate is 0.4.

After the convolution-max-pooling blocks is the classifier block, which contains a flattening layer, two dense layers, and a classifier layer. The first dense layer contains 64 nodes, and the second dense layer contains 1 node. The classifier layer is a dense layer with an output node for the label. SoftMax is used for the activation function. The loss function is binary cross-entropy, and the network optimizer is the Adam optimizer. We use validation set for choosing the best optimizer and for the CNN model in genomic model, Adam optimizer seems the best option.

## Data Availability

The datasets used in the analysis of the method are publicly available. The WISDM and HAR activity recognition datasets are available from the UC Irvine Machine Learning Repository at https://archive.ics.uci.edu/dataset/507 and https://archive.ics.uci.edu/dataset/240, respectively. The Amazon, IMDB and Yelp sentiment analysis datasets are available from GitHub at https://github.com/tarunsharma87/sentiment-analysis-python. The Gene Promoter dataset is available from the UC Irvine Machine Learning Repository at https://archive.ics.uci.edu/dataset/67. The hybrid method source code is available at https://github.com/skinnerlab/DL-ML-Hybrid and https://github.com/holderlb/DL-ML-Hybrid.

## Abbreviations

ML: Machine learning
DL: Deep learning
DNNs: Deep neural networks
HAR: Human activity recognition
CNN: Convolutional neural network
SVM: Support vector machine
VGG: Visual geometry group
UCI: University of California at Irvine
IMDb: Internet movie database
DNN: Deep neural network
NLP: Natural language processing
